# Melatonin improves postprandial hypoglycemia: an unusual presentation of insulinoma

**DOI:** 10.1210/jcemcr/luag067

**Published:** 2026-04-24

**Authors:** Thanh Nguyen, Aleksey Matveyenko, Galina Smushkin

**Affiliations:** Department of Physiology and Biomedical Engineering, Mayo Clinic School of Medicine, Rochester, MN 55902, USA; Department of Physiology and Biomedical Engineering, Mayo Clinic School of Medicine, Rochester, MN 55902, USA; Division of Endocrinology, Metabolism, Diabetes, and Nutrition, Mayo Clinic School of Medicine, Rochester, MN 55902, USA; Department of Medicine, Division of Endocrinology, University of British Columbia, Victoria, BC V5Z 1M9, Canada

**Keywords:** insulinoma, postprandial hypoglycemia, melatonin, melatonin receptors

## Abstract

Insulinoma is the most prevalent neuroendocrine tumor of the pancreas, presenting with a spectrum of neuroglycopenic symptoms. This case report describes a 64-year-old woman with primarily postprandial hypoglycemia, resulting in altered consciousness and confusion occurring several hours after meals. Continuous glucose monitoring (CGM) was used to establish the postprandial pattern of the patient's hypoglycemia, and this facilitated the confirmatory biochemical evaluation by a single blood draw done at the time of symptoms, without performing a 72-hour fast. Imaging demonstrated an 11-mm tumor in the pancreas, which was removed and confirmed to be a well-differentiated neuroendocrine tumor on histopathology. While awaiting pancreatic surgery, the patient began taking melatonin for sleep and reported an improvement in the frequency and severity of hypoglycemia symptoms, which was supported by CGM data and a robust expression of melatonin receptors on the excised tumor. This case raises interest in the role of melatonin receptor signaling in counteracting hyperinsulinemia and corresponding hypoglycemia in patients with insulinoma.

## Introduction

Insulinoma is the most common functioning neuroendocrine tumor of the pancreas, with an estimated incidence of 1 to 4 per 1 000 000 persons per year [1]. Patients present with episodic neuroglycopenic symptoms, including disorientation, confusion, slurred speech, vision changes, and at worst seizures or loss of consciousness. Although fasting hypoglycemia has historically been considered the hallmark of insulinoma, a large retrospective analysis reported that 21% of insulinoma cases present with a mixture of fasting and postprandial hypoglycemia and 6% present with postprandial hypoglycemia alone [1]. The mechanisms by which some insulinomas cause exclusively postprandial hypoglycemia have not been elucidated.

We report a case of insulinoma associated with primarily postprandial hypoglycemia, where the severity of hypoglycemia improved with an over-the-counter melatonin supplement, which the patient serendipitously took for insomnia.

## Case presentation

A 64-year-old woman without a history of diabetes presented for an evaluation of an episode of altered consciousness and slurred speech associated with hypoglycemia. She had a 5-year duration of episodic symptoms of blurry vision and exhaustion, occurring mostly in the afternoons 2 to 3 hours after lunch and improving with food intake. She had been assessed by neurology, with the working diagnosis of atypical migraines. On 2 separate occasions 2 years apart, her spouse had called emergency medical services because he found her confused with slurred speech. Point-of-care glucose was 59 and 31 mg/dL (SI: 3.3 and 1.7 mmol/L) (reference range, 70-198 mg/dL [SI: 3.9-11 mmol/L]). On the first occasion, she was brought to the emergency room after intravenous glucose resuscitation and underwent head imaging, which was unremarkable. She was monitored for 6 hours, during which she maintained normal glucose and had no neurologic symptoms. She was then advised to follow up with neurology and discharged. During the second episode, she was resuscitated with glucose in the field but declined to be taken to the hospital. She began wearing a continuous glucose monitor (CGM) and brought this data when she presented for endocrine evaluation. Her body mass index was 23.5 and she was not taking any prescription medications. Her over-the-counter supplements included vitamin C, vitamin D, vitamin B50 and B2, coenzyme Q10, calcium with magnesium, and omega-3. To manage her symptoms, she had been following a balanced low-glycemic index diet with multiple small meals per day.

## Diagnostic assessment

At the initial endocrine assessment, her 14-day Libre 2 data demonstrated 88% sensor use, 40% below 70 mg/dL (SI: 3.9 mmol/L), with 19% below 54 mg/dL (SI: 3.0 mmol/L). Glucose was generally higher than 70 mg/dL (SI: 3.9 mmol/L) overnight and until 11 Am, with hypoglycemia occurring in the afternoons and evenings. Hyperinsulinemic hypoglycemia was confirmed with a venous blood draw in the afternoon, at the time of symptoms, where glucose was 22 mg/dL (SI: 1.2 mmol/L), C-peptide concentration was 1.9 ng/mL (SI: 634 pmol/L) (reference range, 1.0-3.3 ng/mL [SI: 325-1090 pmol/L]), and insulin concentration was 4.6 µIU/mL (SI: 32 pmol/L) (reference range, <13.7 µIU/mL [SI: <95 pmol/L]). A 72-hour fast was not performed for diagnosis, as the Whipple triad was achieved during the outpatient blood draw. Her past lab work demonstrated several normal-range fasting glucose concentrations previously; a recent fasting glucose was 97 mg/dL (SI: 5.4 mmol/L) with insulin concentration 2.7 µIU/mL (SI: 19 pmol/L).

Computed tomography (CT) and magnetic resonance imaging (MRI) both demonstrated an 11-mm mass in the tail of the pancreas, posterior to the pancreatic duct, with enhanced characteristics consistent with a neuroendocrine tumor.

## Treatment

**Figure 1:**
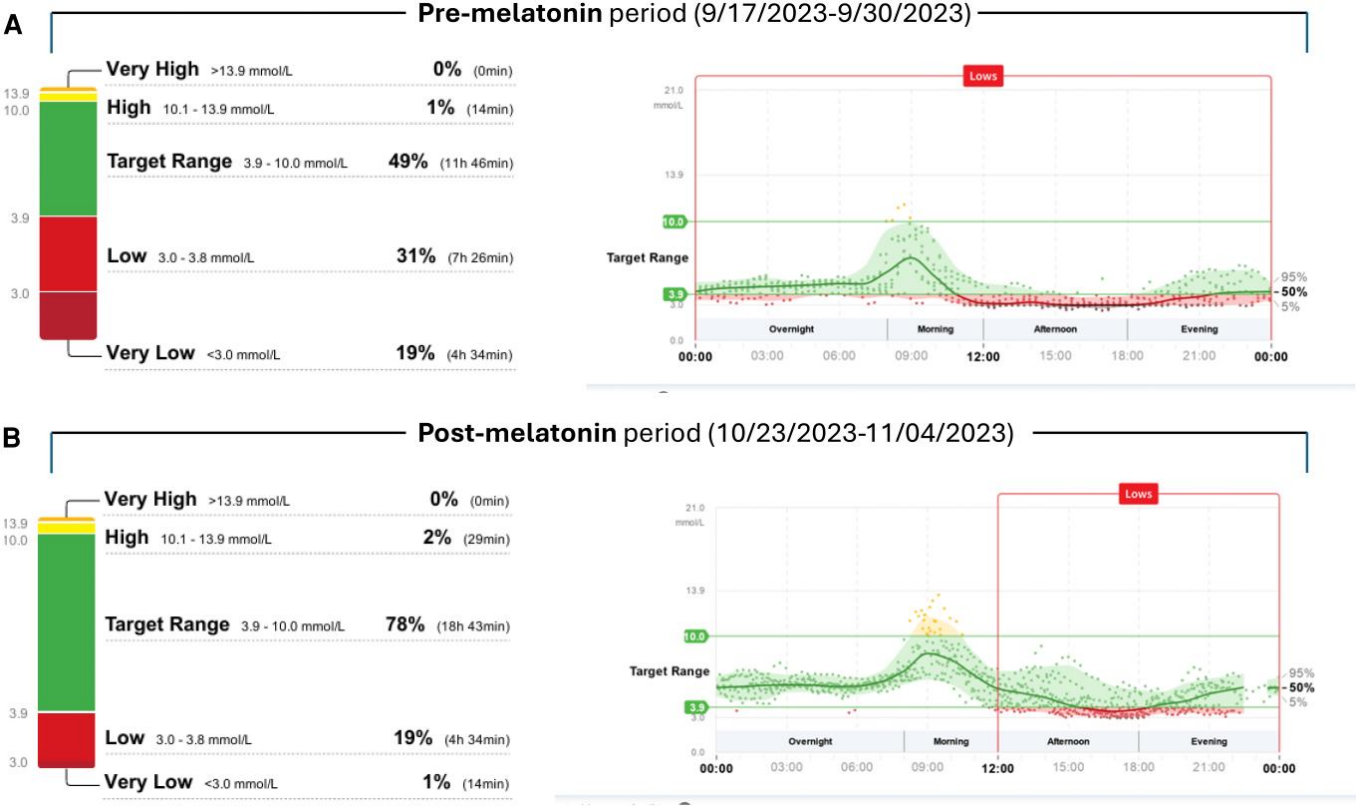
Continuous glucose monitoring (CGM) data in the patient prior and during melatonin supplementation. 14-day glucose data and ambulatory glucose profiles obtained using the FreeStyle Libre system prior (A) and during (B) daily melatonin supplementation in a patient with insulinoma.

The patient was referred for pancreatic surgery. As is common in the Canadian health care system, there was a significant wait time for a surgery date. In the 4 months that she waited to undergo surgery, she began taking an over-the-counter melatonin supplement 10 mg daily at bedtime for insomnia. Surprisingly, she noted a significant improvement in the severity of her hypoglycemia symptoms, as supported by her Libre 2 CGM data, which she reported to her endocrinologist during a follow-up visit. In the 14 days preceding the start of melatonin, she spent 31% of time in the glucose range 54 to 69 mg/dL (SI: 3.0-3.8 mmol/L) and 19% of time in the range below 54 mg/dL (SI: 3.0 mmol/L), whereas in the 14 days on melatonin, she spent 19% of time in the range 54 to 69 mg/dL (SI: 3.0-3.8 mmol/L) and only 1% of time in the range below 54 mg/dL (SI: 3.0 mmol/L). She continued to use melatonin and in the 30 days before surgery, she had spent only 7% of time in the range 54 to 69 mg/dL (SI: 3.0-3.8 mmol/L) and 1% of time below 54 mg/dL (SI: 3.0 mmol/L). Figure 1 depicts the 14-day Libre glucose data and ambulatory glucose profile pre- and post-melatonin.

**Figure 2:**
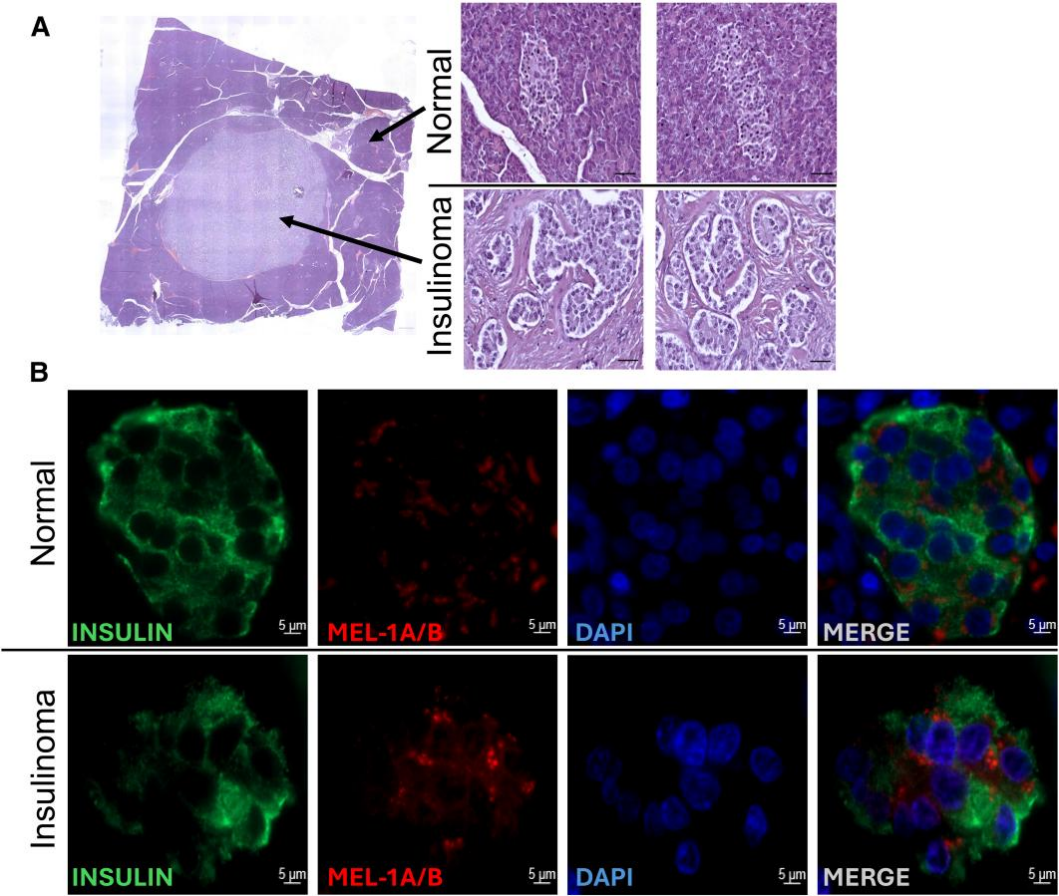
Histopathology of the pancreatic neuroendocrine tumor. (A) Hematoxylin and eosin staining, 5× (left) and 20× (right) of the tumor and the unaffected islets. (B) Representative examples of patient's insulinoma tumor tissue and the unaffected islets stained by immunofluorescence for pan-melatonin receptor antibody (MEL-1A/B, red), insulin (green), and nuclear marker DAPI (blue) imaged at 60× magnification (scale bars, 5 μm).

She underwent a laparoscopic distal pancreatectomy and splenectomy without complications. Histopathology revealed a unifocal, 1-cm well-differentiated neuroendocrine tumor, with Ki-67 of less than 3%, negative margins, without lymphovascular or perineural invasion (Fig. 2A).

## Outcome and follow-up

Immediately postoperatively, the patient experienced hyperglycemia to a glucose level of 270 mg/dL (SI: 15 mmol/L) and required intravenous insulin briefly. She was discharged on no medication. She had discontinued melatonin preoperatively. At last follow-up, 6 weeks postoperative, she was asymptomatic and CGM reported 2% time in the glucose range 54 to 69 mg/dL (SI: 3.0-3.8 mmol/L) without any associated symptoms.

## Discussion

Two aspects of this case are particularly interesting. The first is that the patient had primarily postprandial hypoglycemia occurring daily in the afternoons but never reported hypoglycemic events overnight or in the morning. This pattern of hypoglycemia had resulted in a delay in her diagnosis, as she was initially repeatedly evaluated with laboratory for fasting glucose and insulin, which were normal. Although CGM should not be used for the diagnosis of hypoglycemia in individuals without diabetes, it has been certainly instrumental in this patient's case in elucidating the unusual pattern of hypoglycemia and providing supportive evidence to her subjective improvement in symptoms with melatonin. In fact, without the CGM data, the patient's report of symptomatic improvement with melatonin could have been easily dismissed as placebo effect or the development of hypoglycemia unawareness due to recurrent hypoglycemia.

The second unique aspect of this case is the responsiveness of the patient's hypoglycemia to melatonin supplementation. Melatonin (5-methoxy-N-acetyltryptamine) is a circulating hormone that is released by the pineal gland, with secretion demonstrating robust circadian rhythm beginning a few hours prior to the onset of the night/dark circadian cycle and peaking between midnight and 4 Am. Melatonin levels demonstrate a precipitous decline after the habitual wake time and display very low levels during the day/light circadian cycle. Melatonin coordinates circadian rhythms and neuroendocrine processes via binding to high affinity G-coupled receptors, melatonin receptor subtypes MEL-1A (encoded by *MTNR1A*) and MEL-1B (encoded by *MTNR1B*) [2]. Melatonin receptors are ubiquitously expressed in the central nervous system and peripheral tissues and have been reported to demonstrate expression in human and rodent pancreatic islets [3, 4]. Melatonin's actions are mediated through activation of a classic Gi (inhibitory)-coupled receptor cascade, which results in the inhibition of adenylyl cyclase (AC) activity and a subsequent reduction in cAMP production leading to decreased activation of downstream targets such as protein kinase A (PKA) [5]. Importantly, the activation of melatonin receptors in pancreatic β-cells in vitro leads to a robust reduction in insulin secretion, and the attenuation of β-cell stress [6-8]. Consistently, acute melatonin supplementation in humans at doses similar to our patient's daily intake (5-10 mg/day) results in attenuated postprandial insulin response and the induction of postprandial hyperglycemia [9].

Suppressive effects of melatonin on insulin secretion in humans suggest that the improvement in the frequency of hypoglycemia with over-the-counter melatonin supplement may be due to the inhibition of insulin secretion via the activation of the melatonin receptors on the tumor. To provide supporting evidence, we performed immunofluorescence analysis of the tumor and the unaffected pancreatic islets for the immunoreactivity to melatonin receptors (MEL-1A/B) (Fig. 2B). Analysis of immunofluorescence staining showed robust, albeit heterogeneous melatonin receptor expression pattern in the tumor tissue. Qualitatively, melatonin receptor expression appears to be enhanced in the tumor compared to the unaffected islets; however, examination of additional insulinoma cases will be required to provide quantitative assessment of insulinoma melatonin receptor expressions compared to unaffected islets. Intriguingly, studies in individuals carrying a common gain-of-function melatonin receptor 2 (MTNR1B) genetic variant report an association between increased MEL-1B expression/function in β cells and a reduction in insulin secretion [7]. Collectively, future studies are warranted to explore whether pharmacological and/or genetic activation of the melatonin receptor signaling plays a protective role to counteract hyperinsulinemia and corresponding hypoglycemia in patients with insulinoma.

## Learning points

- Some insulinomas present with primarily postprandial symptoms, where measurement of a fasting glucose and insulin concentrations are unrevealing, resulting in a delay of diagnosis.
- While CGM should not be used to diagnose a hypoglycemic disorder, due to a reduced accuracy in the hypoglycemia range, it is a valuable tool for detecting a pattern of hypoglycemia, enabling coordination of the timing of a random venous blood glucose draw at the laboratory for hypoglycemia confirmation and workup.
- Melatonin supplementation may improve hyperinsulinemic hypoglycemia related to an insulinoma, and studies of insulinoma responsiveness to melatonin are needed.

## Data Availability

Data sharing is not applicable to this article as no datasets were generated or analyzed during the current study.

## Abbreviations

CGM: continuous glucose monitoring
MEL-1B: melatonin receptor 1B
